# Efficacy of Antibodies Targeting TfR1 in Xenograft Mouse Models of AIDS-Related Non-Hodgkin Lymphoma

**DOI:** 10.3390/cancers15061816

**Published:** 2023-03-17

**Authors:** Tracy R. Daniels-Wells, Pierre V. Candelaria, Emiko Kranz, Jing Wen, Lan Wang, Masakazu Kamata, Juan C. Almagro, Otoniel Martínez-Maza, Manuel L. Penichet

**Affiliations:** 1 Division of Surgical Oncology, Department of Surgery, David Geffen School of Medicine, University of California, Los Angeles (UCLA), Los Angeles, CA 90095, USA; 2 Department of Microbiology, Immunology, and Molecular Genetics, David Geffen School of Medicine, University of California, Los Angeles (UCLA), Los Angeles, CA 90095, USA; 3 UCLA AIDS Institute, Los Angeles, CA 90095, USA; 4 Division of Hematology and Oncology, David Geffen School of Medicine, University of California, Los Angeles (UCLA), Los Angeles, CA 90095, USA; 5 UCLA Jonsson Comprehensive Cancer Center, Los Angeles, CA 90095, USA; 6 Department of Microbiology, Heersink School of Medicine, University of Alabama, Birmingham, AL 35294, USA; 7 GlobalBio, Inc., Cambridge, MA 02138, USA; 8 Department of Obstetrics and Gynecology, David Geffen School of Medicine, University of California, Los Angeles (UCLA), Los Angeles, CA 90095, USA; 9 Department of Epidemiology, UCLA Fielding School of Public Health, Los Angeles, CA 90095, USA; 10 The Molecular Biology Institute, University of California, Los Angeles (UCLA), Los Angeles, CA 90095, USA

**Keywords:** AIDS-NHL, antibody therapy, Burkitt lymphoma, CD71, non-Hodgkin lymphoma, transferrin receptor

## Abstract

**Simple Summary:**

There is an increased risk for the development of certain cancers, including non-Hodgkin lymphoma (NHL), in individuals infected with the human immunodeficiency virus (HIV). Therefore, NHL is considered to be an acquired immunodeficiency syndrome (AIDS)-related malignancy (AIDS-NHL). There are several subtypes of NHL, but the majority of these cancers are of B-cell origin. HIV infection leads to the chronic activation of B cells, which results in high expression of a protein called the transferrin receptor 1 (TfR1), B-cell dysfunction, and ultimately the development of AIDS-NHL. TfR1 is responsible for iron uptake by cells. Iron is needed for multiple cellular processes, including DNA synthesis, which is required for cell proliferation. Thus, targeting this receptor is a meaningful strategy for treatment of AIDS-NHL. This study shows that antibodies targeting TfR1 prolong survival in two mouse models of AIDS-NHL, suggesting that these antibodies have potential as a therapy for AIDS-NHL.

**Abstract:**

Transferrin receptor 1 (TfR1), also known as CD71, is a transmembrane protein involved in the cellular uptake of iron and the regulation of cell growth. This receptor is expressed at low levels on a variety of normal cells, but is upregulated on cells with a high rate of proliferation, including malignant cells and activated immune cells. Infection with the human immunodeficiency virus (HIV) leads to the chronic activation of B cells, resulting in high expression of TfR1, B-cell dysfunction, and ultimately the development of acquired immunodeficiency syndrome-related B-cell non-Hodgkin lymphoma (AIDS-NHL). Importantly, TfR1 expression is correlated with the stage and prognosis of NHL. Thus, it is a meaningful target for antibody-based NHL therapy. We previously developed a mouse/human chimeric IgG3 specific for TfR1 (ch128.1/IgG3) and showed that this antibody exhibits antitumor activity in an in vivo model of AIDS-NHL using NOD-SCID mice challenged intraperitoneally with 2F7 human Burkitt lymphoma (BL) cells that harbor the Epstein-Barr virus (EBV). We have also developed an IgG1 version of ch128.1 that shows significant antitumor activity in SCID-Beige mouse models of disseminated multiple myeloma, another B-cell malignancy. Here, we aim to explore the utility of ch128.1/IgG1 and its humanized version (hu128.1) in mouse models of AIDS-NHL. To accomplish this goal, we used the 2F7 cell line variant 2F7-BR44, which is more aggressive than the parental cell line and forms metastases in the brain of mice after systemic (intravenous) administration. We also used the human BL cell line JB, which in contrast to 2F7, is EBV-negative, allowing us to study both EBV-infected and non-infected NHL tumors. Treatment with ch128.1/IgG1 or hu128.1 of SCID-Beige mice challenged locally (subcutaneously) with 2F7-BR44 or JB cells results in significant antitumor activity against different stages of disease. Treatment of mice challenged systemically (intravenously) with either 2F7-BR44 or JB cells also showed significant antitumor activity, including long-term survival. Taken together, our results suggest that targeting TfR1 with antibodies, such as ch128.1/IgG1 or hu128.1, has potential as an effective therapy for AIDS-NHL.

## 1. Introduction

Infection with the human immunodeficiency virus (HIV) has long been known to be associated with a marked increase in risk for B-cell lymphoma [1,2,3,4,5]. In fact, acquired immunodeficiency syndrome-associated non-Hodgkin lymphoma (AIDS-NHL) has been an AIDS-defining condition since 1985 [6]. There are several subtypes of AIDS-NHL, including Burkitt lymphoma (BL) and diffuse large B-cell lymphoma (DLBCL), but the great majority of AIDS-NHL are of B-cell origin [2,7]. AIDS-NHL remains a significant clinical problem in the era of combination antiretroviral therapy (cART). While an overall decrease in the incidence of AIDS-NHL has been observed in the cART era, the risk for AIDS-NHL remains elevated [2,3,5]. Additionally, not all AIDS-NHL subtypes have decreased in incidence in the cART era. For example, AIDS-BL has been less affected by cART than that of other NHL subtypes [3,5,8,9]. In fact, AIDS-NHL is the most common AIDS-related cancer in developed countries, and it continues to have a significant clinical impact, accounting for 20–30% of all AIDS-related causes of death [3,5,10,11].

HIV infection leads to the chronic activation of B cells, resulting in high expression of the transferrin receptor 1 (TfR1, also known as CD71), B-cell dysfunction, and ultimately the development of AIDS-NHL [12,13,14,15]. TfR1 is a type II transmembrane protein that is critical for cellular iron uptake through its interaction with iron-bound transferrin [16]. Iron is a co-factor for several cellular enzymes, such as ribonucleotide reductase that is required for DNA synthesis and thus cellular proliferation [16]. TfR1 is expressed at low levels on most normal cells and at higher levels on cells with a high rate of proliferation (such as activated immune cells) or a high need for iron (such as erythroid progenitors) [16]. In general, malignant cells overexpress TfR1, resulting in its identification as a universal cancer marker [16,17], and both TfR1 and iron have been implicated in cancer cell survival [16]. The overexpression of TfR1 is associated with poor prognosis or more aggressive disease in several types of cancer, including various solid cancers and hematopoietic malignancies (such as NHL) [16]. Interestingly, patients infected with HIV often develop more aggressive NHL that express even higher levels of TfR1 messenger RNA compared to NHL cells from the general population [18,19]. Taken together, these observations indicate that TfR1 is a meaningful target for AIDS-NHL antibody-mediated cancer therapy.

We have previously developed a mouse/human chimeric IgG3 antibody specific for TfR1 (ch128.1/IgG3) that does not cross-react with TfR2, nor does it interfere with the binding of the TfR1 ligands transferrin or the hemochromatosis protein (HFE) to the receptor [20,21,22]. This IgG3 antibody exhibits antitumor activity in mouse models of disseminated human multiple myeloma (MM) using SCID-Beige mice challenged intravenously (i.v.) with KMS-11 or ARH-77 cells [23,24] and in a mouse model of AIDS-NHL using NOD-SCID mice challenged intraperitoneally (i.p.) with 2F7 human BL cells [25]. More recently, we have also developed a mouse/human chimeric IgG1 (ch128.1/IgG1) and shown antitumor activity of this antibody in disseminated xenograft mouse models of MM using SCID-Beige mice and human KMS-11, MM.1S, or MM.1R cells [26]. We now aim to evaluate, for the first time, the antitumor activity of ch128.1/IgG1 and its newly developed humanized version (hu128.1) in two xenograft mouse models of AIDS-NHL.

Epstein-Barr virus (EBV) is a (B-cell)-tropic, human γ-herpesvirus that has significant oncogenic potential [27]. Ongoing control of EBV infection requires a vigorous, life-long immune response. Therefore, EBV-associated cancers are a particular problem in immunodeficient individuals, including those who are infected with HIV [27]. While EBV infection of tumor cells is clearly associated with some AIDS-NHL, many AIDS-NHL are not infected with EBV. Thus, it is important to have models that mimic both EBV-associated and EBV-negative AIDS-NHL. However, cell lines that model EBV-negative tumors are rare [28]. For our studies, we used two AIDS-NHL cell lines, the 2F7-BR44 cell line, which is positive for EBV, and the JB cell line, which is unique in that it is negative for EBV.

## 2. Materials and Methods

### 2.1. Cell Lines

2F7-BR44 human BL cells are EBV-positive and were previously described [29]. These cells were isolated from a mouse brain metastatic site after i.p. administration of 2F7 cells (from ATCC, Rockville, MD; CRL-10237-discontinued) and adapted to cell culture. The original 2F7 cell line was derived from an AIDS-associated B-cell NHL. 2F7-BR44 cells express firefly luciferase and were grown in Iscove’s modified Dulbecco’s medium (IMDM) supplemented with 15% fetal bovine serum (FBS; R&D Systems, Minneapolis, MN, USA), 50 μg/mL zeocin (Thermo Fisher Scientific, Waltham, MA, USA), and penicillin/streptomycin (Thermo Fisher Scientific) in 5% CO_2_ at 37 °C. The human BL cell line JB was a kind gift from Dr. Ashlee V. Moses (Oregon Health and Science University, Portland, OR, USA) [28]. These cells were previously described and originated from the culture of microvascular endothelial cell (MVEC) stroma derived from an AIDS patient with BL cells, which resulted in the spontaneous and sustained growth of the BL cells (named JB) [28]. JB cells are EBV-negative [28] and were grown in IMDM supplemented with 15% FBS and penicillin/streptomycin in 5% CO_2_ at 37 °C.

### 2.2. Recombinant Antibodies

The construction and production of ch128.1/IgG1, a mouse/human chimeric antibody targeting TfR1 that contains the variable regions of the 128.1 murine IgG1 antibody [22], in NS0/1 murine myeloma cells have been described previously [26]. In order to further the development of ch128.1/IgG1 for therapeutic use in humans, this antibody was humanized by complementarity-determining region (CDR) grafting [30]. Similar to the parental chimeric antibody ch128.1, the humanized antibody (hu128.1) contains human γ1 and κ constant regions. The resultant variable regions contain human framework residues with backmutations while maintaining the mouse CDRs [30]. The hu128.1 was produced in Chinese hamster ovary (CHO) cells by Evitria AG (Zürich-Schlieren, Switzerland) [30]. In the present studies, we used the hu128.1 variant H6L7, which shares the binding properties of ch128.1/IgG1 and exhibits superior thermal stability [30]. A mouse/human chimeric IgG1/κ antibody specific for the hapten dansyl (5-dimethylamino naphthalene-1-sulfonyl chloride) was used as an isotype negative control antibody and was produced in murine myeloma cells [31].

### 2.3. In Vivo Efficacy Studies

All animal research was approved by the UCLA Chancellor’s Animal Research Committee (Institutional Animal Care and Use Committee) and was conducted in accordance with guidelines for the housing and care of laboratory animals of the National Institutes of Health and Association for Assessment and Accreditation of Laboratory Animal Care International. C.B-17 SCID-Beige female mice (8–12 weeks old) were obtained and housed in the Defined Flora Mouse Core Facility in the Department of Radiation Oncology at UCLA. To establish local tumors, 2 × 10^6^ 2F7-BR44 or 5 × 10^6^ JB cells per mouse were administered subcutaneously (s.c.) in Hank’s Balanced Salt Solution (HBSS; Thermo Fisher Scientific) into the right flank. Mice were then treated i.v. via the tail vein at various times with 400 μg per mouse of ch128.1/IgG1, hu128.1, or the IgG1 negative control antibody in phosphate-buffered saline (PBS, Thermo Fisher Scientific) as indicated in each figure. For treatments on Day 2 after tumor challenge, mice were randomized into treatment groups. For treatments that started after tumors developed (when tumors measured ~3–5 mm in diameter), mice were distributed into equal groups based on calculated tumor volumes so that all groups had similar average tumor volumes. Tumor growth was measured with a caliper and tumor volumes were calculated as (length × width^2^)/2. Tumor growth rate graphs were generated using GraphPad Prism, Version 9 (GraphPad Software Inc., La Jolla, CA, USA). Animal survival was determined as the time from tumor challenge until the tumor reached 1.5 cm in diameter. Statistical analysis of tumor volumes in each group was performed using the rate-based T/C method [32]. Graphs of individual growth curves are included in Supplementary Figures S1–S6. To establish disseminated disease, 5 × 10^6^ 2F7-BR44 or JB cells were injected i.v. in HBSS via the tail vein. Mice were treated 2 days later i.v. via the tail vein with 100 μg per mouse of ch128.1/IgG1, hu128.1, or the IgG1 negative control antibody in PBS as indicated in each figure. Animal survival was determined as the time from tumor challenge until mice became moribund or developed hind-limb paralysis. In some studies, mice were inoculated with PBS alone (buffer) as an additional negative control group. All survival plots, median survival, and statistical analysis (log-rank test) were generated or determined using GraphPad Prism. 

Antibody dosing for disseminated 2F7-BR44 and JB models was based on previous studies using ch128.1/IgG1 in disseminated xenograft models of human MM, another B-cell malignancy, using SCID-Beige mice [26]. Treatments were started on Day 2 for studies using disseminated models for us to compare with previous results in disseminated MM models. Additionally, disseminated models are more aggressive, especially those using the 2F7-BR44 cell line, which has been shown to be highly metastatic [29]. Dosing for models where cells are injected s.c. was increased to 400 μg per mouse since the tumors grow as large masses and are generally more difficult for therapeutic antibodies to penetrate. These studies were initially conducted with treatment starting once tumors grew to determine if our antibodies had efficacy against established tumors. Studies were also conducted with treatment given on Day 2 to model a low-tumor burden or micrometastatic disease setting, which is relevant in human disease since this often occurs after surgical removal of the primary tumor. Micrometastatic disease is often difficult to treat since the location of the tumor is unknown. It is important to evaluate antibody efficacy for all disease settings. For each model (depending on cell line and route of cell injection), each set of studies was performed at least twice for each treatment schedule.

### 2.4. Bioluminescence Imaging

2F7-BR44 distribution in the animals was monitored weekly after tumor challenge by bioluminescence imaging using an IVIS Lumina II In Vivo Imaging System (PerkinElmer, Waltham, MA, USA). Pierce^TM^ D-luciferin (4.5 mg; Thermo Fisher Scientific) was injected s.c. in the abdominal area and imaging performed after 5–10 min under isoflurane anesthesia. Tumor burden was quantified as the total photon flux per second within the region of interest (whole body of the mouse) using the Living Image^®^ Software, Version 4.7.3 (PerkinElmer). All regions of interest were identical in size, and sensitivity settings were adjusted to maintain the same counts per pixel (200–800 for Week 1 images and 10,000–30,000 for Week 3 images in the local tumor model; 2000–12,000 for the disseminated model). Imaging was performed in the UCLA Crump Preclinical Imaging Technology Center.

## 3. Results

### 3.1. Efficacy of ch128.1/IgG1 and hu128.1 in a Local Model of AIDS-NHL Using 2F7-BR44 Cells

**Figure 1:**
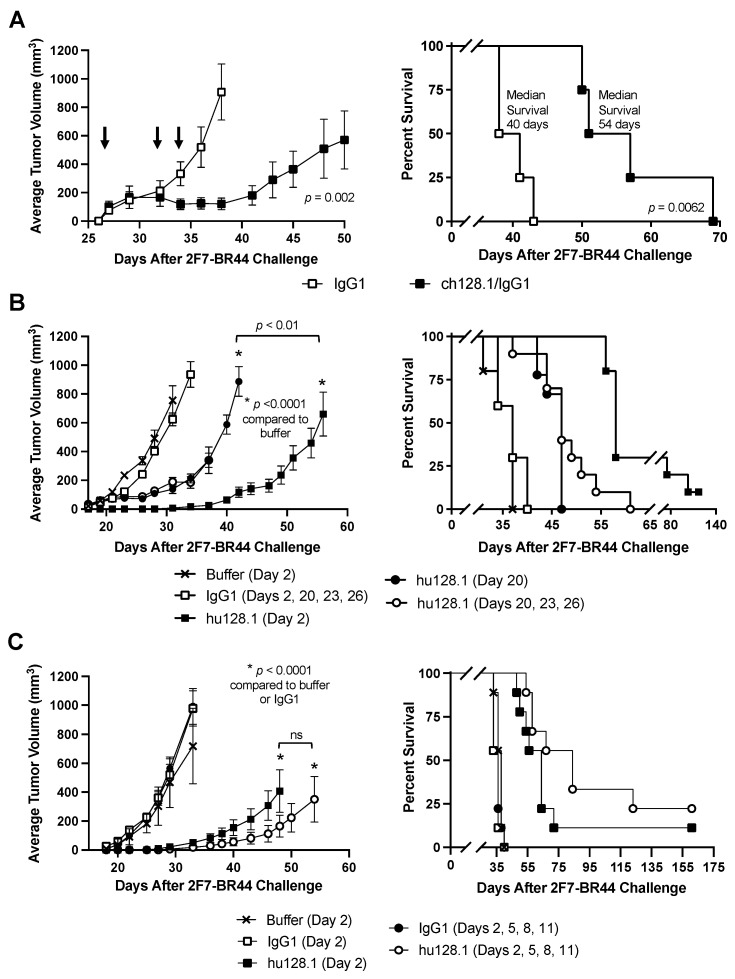
Local model of AIDS-NHL using 2F7-BR44 cells. (**A**) SCID-Beige mice were challenged s.c. with 2 × 10^6^ cells in the right flank. Once all mice developed palpable tumors (Day 27), mice were distributed by tumor size into two equal groups (*n* = 4 per group). Mice were treated i.v. with either 400 μg isotype negative control antibody (IgG1) or ch128.1/IgG1 on Days 27, 32, and 34 (indicated by arrows). The tumor growth curves (left panel) and Kaplan-Meier survival plot (right panel) are shown. (**B**,**C**) Mice were treated i.v. with 400 μg of the antibodies as indicated (*n* = 9 or 10 per group). The tumor growth curves (left panels) and Kaplan-Meier survival plots (right panels) are shown. Median survival and the results of the statistical analysis for panels B and C are shown in Table 1. For all panels, tumor growth rates are shown as the average tumor volume with the standard error of the mean (SEM) for each treatment group. Growth curves reflect the time when all animals in the treatment group were alive. Individual tumor growth curves are shown in Supplementary Figures S1–S3. ns, not significant.

To explore the potential of targeting TfR1 for AIDS-NHL treatment, we sought to evaluate the efficacy of ch128.1/IgG1 or hu128.1 in two new xenograft mouse models of AIDS-NHL. We established local models of disease as well as disseminated models, so cells were injected either s.c. or i.v., respectively. We first used 2F7-BR44 cells that were isolated previously based on their ability to metastasize to the brain upon i.p. administration into the mouse [29]. In our first study in the local disease model (cells were administered s.c.), treatment was initiated when all animals developed measurable tumors (approximately 3–5 mm in diameter), a late-stage tumor model. Animals treated three times with 400 μg of ch128.1/IgG1 showed decreased tumor growth and prolonged survival compared to animals treated with the IgG1 control antibody (Figure 1A and Figure S1). However, all animals eventually succumbed to the disease. 

This study was repeated with a larger number of animals and to also include groups where mice were treated on Day 2 after tumor challenge to simulate an early-stage (or low tumor burden) model. Mice treated three times with 400 μg of hu128.1 once tumors had developed, again showed delayed tumor growth and prolonged survival (Figure 1B and Figure S2) similar to ch128.1/IgG1 (Figure 1A). When mice were treated on Day 2, hu128.1 showed an even greater antitumor effect, which is expected due to the decreased tumor burden at the time of treatment. The median survivals for each group and the results of the statistical analysis are shown in Table 1. One animal out of 10 treated with hu128.1 on Day 2 did not develop a tumor. 

In the third study for this local disease model using 2F7-BR44 cells, we aimed to determine if multiple treatments of 400 μg starting on Day 2 could block tumor formation. Mice given one treatment of hu128.1 on Day 2 again delayed tumor growth and prolonged survival compared to mice treated with the IgG1 control antibody or buffer alone on Day 2 only (Figure 1C and and Figure S3). The median survivals for each group and the results of the statistical analysis are shown in Table 1. Mice treated four times with hu128.1 (on Days 2, 5, 8, and 11) showed an increased median survival and delayed tumor growth compared to one treatment on Day 2 alone, but the differences were not statistically significant. In this study, one out of nine mice treated with hu128.1 on Day 2 did not develop a tumor, while two out of nine animals treated four times with hu128.1 never developed tumors.

**Figure 2:**
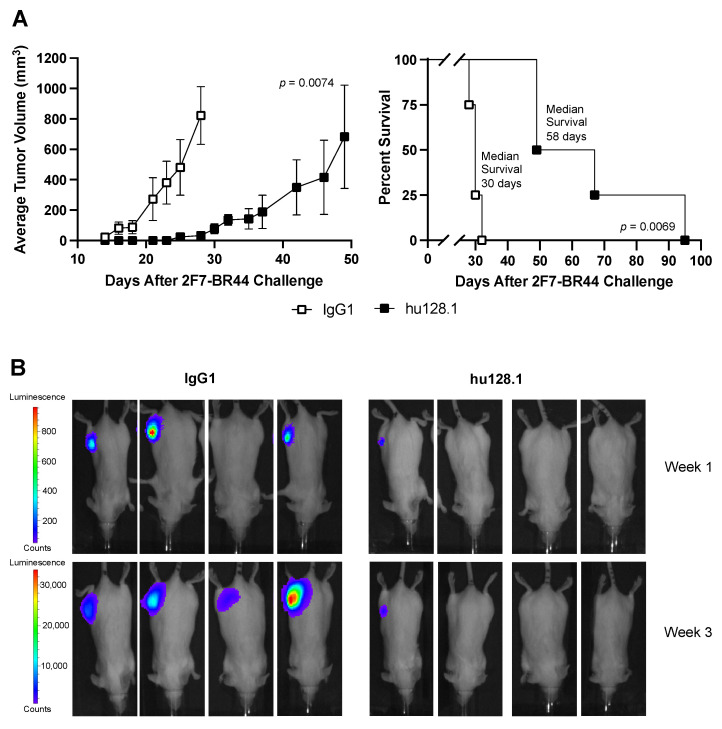
Bioluminescence imaging of mice in the local 2F7-BR44 model. SCID-Beige mice were challenged s.c with 2 × 10^6^ 2F7-BR44 cells in the right flank. Two days later mice were treated i.v. with 400 μg isotype negative control antibody (IgG1) or hu128.1 (*n* = 4 per group). (**A**) The tumor growth curves (left panel) and Kaplan-Meier survival plot (right panel) are shown. Tumor growth rates are shown as the average tumor volume with the SEM for each treatment group. Growth curves reflect the time when all animals in the treatment group were alive. Individual tumor growth curves are shown in Supplementary Figure S4. (**B**) Dorsal bioluminescence images for Weeks 1 and 3 after tumor challenge are shown for each mouse.

Tumor localization in mice was determined using bioluminescence imaging. For this study, mice were treated with hu128.1 on Day 2 after tumor challenge. Imaging was conducted every week while control animals (treated with the IgG1 control antibody) were still alive. In this study, 400 μg hu128.1 again slowed tumor growth and prolonged animal survival (Figure 2A and Figure S4). Additionally, 2F7-BR44 tumors were not detected outside of the flank in control mice during the imaging time frame (Figure 2B). Therefore, these cells, implanted s.c., did not appear to spontaneously metastasize in this mouse model. Taken together, both anti-TfR1 antibodies demonstrate the ability to slow tumor growth and prolong survival of mice bearing local 2F7-BR44 tumors. 

**Table 1:** Median survival and statistical analysis for the data in the local model of AIDS-NHL using 2F7-BR44 cells.

Treatment	Number of Animals	Median Survival (Days)	*p*-Value Compared to IgG1	*p*-Value Compared to Buffer	*p*-Value Compared to hu128.1 (Day 2)
**Data presented in Figure 1B**					
Buffer (Day 2)	10	37			
400 μg IgG1 (Days 2, 20, 23, 26)	10	37		0.2094	
400 μg hu128.1 (Day 2)	10	58	<0.0001	<0.0001	
400 μg hu128.1 (Day 20)	9	47	<0.0001	<0.0001	<0.0001
400 μg hu128.1 (Days 20, 23, 26)	10	47	<0.0001	<0.0001	0.0003
**Data presented in Figure 1C**					
Buffer (Day 2)	9	38			
400 μg IgG1 (Day 2)	9	36		0.1307	
400 μg hu128.1 (Day 2)	9	64	<0.0001	<0.0001	
400 μg IgG1 (Days 2, 5, 8, 11)	9	36		0.2262	
400 μg hu128.1 (Days 2, 5, 8, 11)	9	84	<0.0001	<0.0001	0.1555

### 3.2. Efficacy of ch128.1/IgG1 and hu128.1 in a Disseminated Model of AIDS-NHL Using 2F7-BR44 Cells

**Figure 3:**
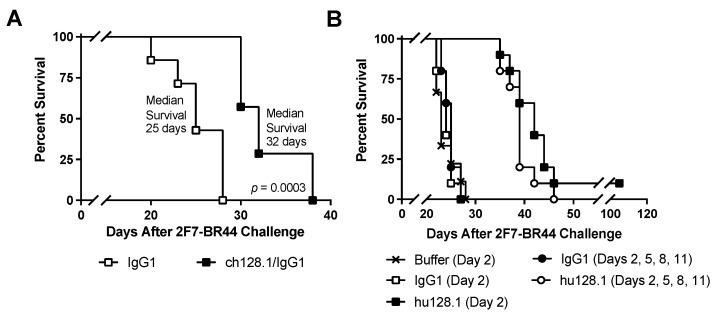
Disseminated model of AIDS-NHL using 2F7-BR44 cells. SCID-Beige mice were challenged i.v with 5 × 10^6^ cells via the tail vein. (**A**) Mice were treated i.v. on Day 2 after tumor challenge with 100 μg isotype negative control antibody (IgG1) or ch128.1/IgG1 (*n* = 7 per group). (**B**) Treatment schedules are indicated. Kaplan-Meier survival plots are shown. Median survival and the results of the statistical analysis for panel B are shown in Table 2 (*n* = 9 or 10 per group).

**Figure 4:**
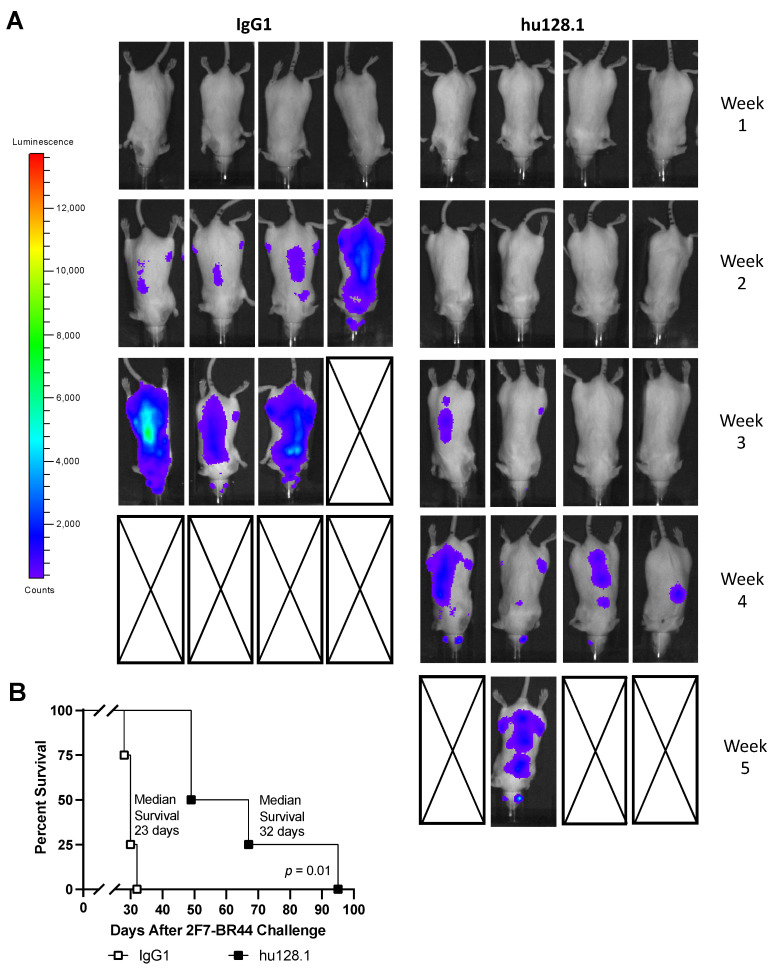
Bioluminescence imaging of mice in the disseminated 2F7-BR44 model. SCID-Beige mice were challenged i.v. with 5 × 10^6^ 2F7-BR44 cells via the tail vein. Two days later mice were treated i.v. with 100 μg isotype negative control antibody (IgG1) or hu128.1 (*n* = 4 per group). (**A**) Bioluminescence was monitored weekly after tumor inoculation. Dorsal images are shown for each mouse. Boxes with a black X represent deceased mice. (**B**) Kaplan-Meier survival plot showing median survival and *p*-value.

For all studies using this disseminated model, mice were challenged with 5 × 10^6^ 2F7-BR44 cells, and treatment with 100 μg ch128.1/IgG1 or hu128.1 began on Day 2. In the first study, a single treatment with ch128.1/IgG1 prolonged survival compared to treatment with the IgG1 control antibody (Figure 3A). A single treatment with hu128.1 also prolonged survival, compared to either the IgG1 control antibody or buffer alone (Figure 3B), in a subsequent study. The median survival for each group and the results of the statistical analysis are shown in Table 2. However, multiple treatments with hu128.1 did not further prolong survival compared to one treatment (Figure 3B and Table 2). Bioluminescence imaging confirmed that 2F7-BR44 injected i.v. causes disseminated disease, including brain metastases (Figure 4A). A single treatment with hu128.1 prolongs survival (Figure 4B) and prevents metastatic growth in the brain, but is not enough to block tumor growth throughout the body (Figure 4A). Together these data show that both ch128.1/IgG1 and hu128.1 has potent antitumor effects in the highly aggressive, disseminated 2F7-BR44 mouse model.

### 3.3. Efficacy of ch128.1/IgG1 and hu128.1 in Local and Disseminated Models of AIDS-NHL Using JB Cells

**Figure 5:**
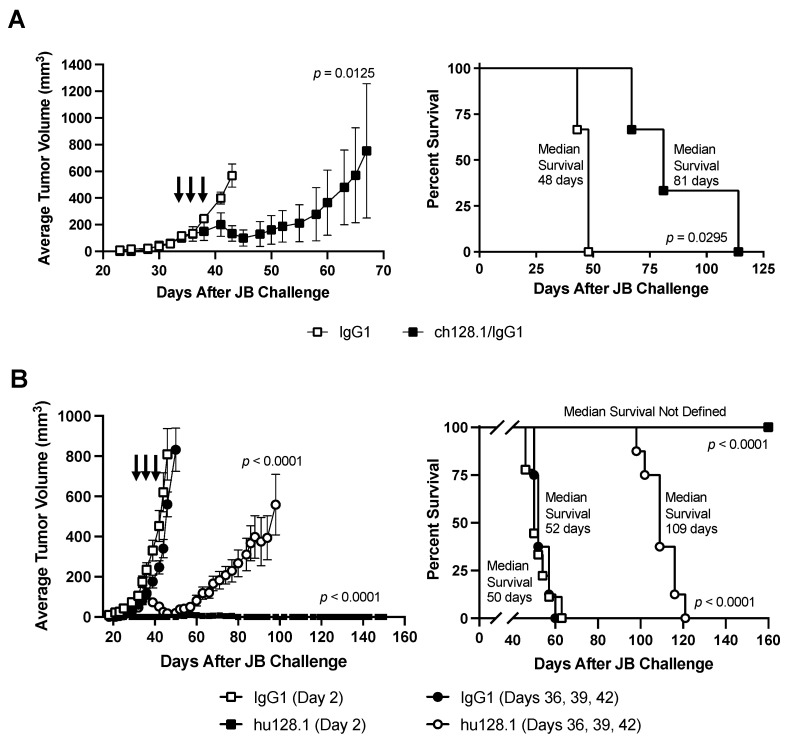
Local model of AIDS-NHL using JB cells. SCID-Beige mice were challenged s.c. with 5 × 10^6^ cells in the right flank. (**A**) Once all mice developed palpable tumors (Day 34), mice were distributed by tumor size into two groups (*n* = 3 per group). Mice were treated i.v. with either 400 μg isotype negative control antibody (IgG1) or ch128.1/IgG1 on Days 34, 36, and 38. The tumor growth curves (left panel) and the Kaplan-Meier survival plot (right panel) are shown. Arrows indicate when treatment was given. Individual tumor growth curves are shown in Supplementary Figure S5. (**B**) Mice were treated with 400 μg isotype negative control antibody (IgG1) or hu128.1 on Day 2, representing an early-stage tumor model (*n* = 9 per group). For a late-stage tumor model, mice that were treated once all developed palpable tumors (*n* = 8 per group). Mice were then grouped by tumor size and treated i.v. with either 400 μg isotype negative control antibody (IgG1) or ch128.1/IgG1 on Days 36, 39, and 42. The tumor growth curves (left panel) and the Kaplan-Meier survival plot (right panel). Arrows indicate when treatment was given. Individual tumor growth curves are shown in Supplementary Figures S5 and S6. For both panels, the growth curves reflect the time when all animals in the treatment group were alive. The tumor growth curves indicate the average tumor volume with the SEM for each treatment group.

Most AIDS-NHL cell lines are positive for EBV. The JB cell line is unique in that it is a BL cell line that is EBV-negative, allowing us to study AIDS-NHL in the absence of infection with this virus. These cells were administered s.c to develop a local xenograft model of AIDS-NHL. In the first pilot study using a small number of mice, treatment was initiated when tumors were palpable (measuring approximately 3–5 mm in diameter) and consisted of three i.v. antibody treatments of 400 μg. Treatment with ch128.1/IgG1 inhibited tumor growth and significantly prolonged the survival compared to mice treated with the IgG1 control antibody ( Figure 5A and Figure S5). However, tumors began to grow after cessation of the treatment, and mice eventually succumbed to the disease. 

We then repeated this study with larger groups of mice and included an early-stage tumor model where mice were treated on Day 2 after JB inoculation. Treatment with hu128.1 after tumors developed (late-stage disease) slowed tumor growth and prolonged survival compared to the IgG1 control antibody-treated mice (Figure 5B and Figure S6), similar to ch128.1/IgG1 (Figure 5A). All mice eventually succumbed to the disease. However, one treatment with 400 μg hu128.1 on Day 2 completely blocked tumor growth, and all mice survived (without tumor development) for the duration of the study (up to 160 days after JB challenge). Mice treated with buffer alone on Day 2 showed similar results to mice treated with the IgG1 control antibody on Day 2 (Figure S7).

**Figure 6:**
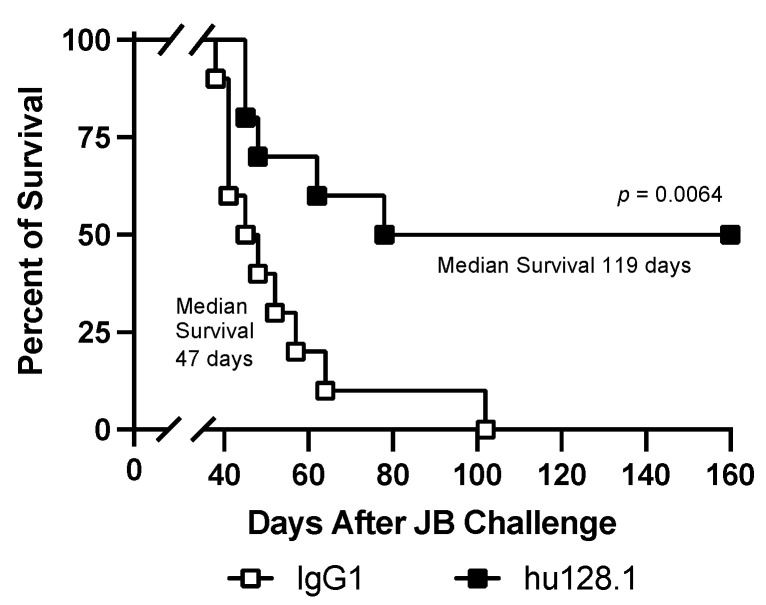
Disseminated model of AIDS-NHL using JB cells. SCID-Beige mice were challenged i.v. with 5 × 10^6^ cells via the tail vein. Mice were treated i.v. on Day 2 after tumor challenge with 100 μg isotype negative control antibody (IgG1) or hu128.1 (*n* = 10 per group). Kaplan-Meier survival plot showing median survival and *p*-value.

JB cells were also inoculated i.v. to create a disseminated AIDS-NHL model. In these studies, mice treated with 100 μg hu128.1 on Day 2 after tumor challenge showed significant prolongation of survival compared to IgG1 control antibody-treated mice (Figure 6). This study was also conducted with ch128.1/IgG1 and a similar prolongation of survival was observed in two independent studies (Figure S8).

## 4. Discussion

AIDS-NHL differ from similar forms of NHL seen in the general population in that a much higher proportion of AIDS-NHL are EBV-positive tumors. Nearly half of AIDS-NHL are EBV-positive, while most non-AIDS-NHL lack EBV [7,12]. Additionally, AIDS-NHL are more often found in extranodal locations and represent more clinically aggressive cancers [7,12]. Ongoing control of EBV infection requires a long-term immune response. Therefore, EBV-associated cancers are a particular problem in immunodeficient individuals, including those who are infected with HIV, or organ-transplant recipients who are immunosuppressed to avoid transplant rejection [27]. Consistent with this, those HIV-infected individuals who have the most profound loss of immunity (e.g., the lowest CD4 numbers) are those who are at greatest risk for the development of EBV-positive NHL, particularly primary central nervous system (CNS) lymphoma, which are virtually all EBV-positive tumors [7,12]. There also may be molecular differences between AIDS-NHL and non-AIDS-associated NHL that are driven by the presence of HIV and/or other oncogenic viruses (such as EBV), but more work is needed to better define such differences [2,7]. Interestingly, we have previously shown that ch128.1/IgG1 can prevent EBV-driven activation and growth of human B-cell tumors in vivo using a NOD/SCID gamma (NSG) immunodeficient mouse model, suggesting that anti-TfR1 antibodies can be used to prevent the development of EBV-associated B-cell malignancies [33]. 

In this study, we demonstrated, for the first time, that both ch128.1/IgG1 and its humanized counterpart (hu128.1) exhibit antitumor activity in models of AIDS-NHL that are EBV-positive (2F7-BR44 cells) or EBV-negative (JB cells). This activity included inhibition of tumor growth and prolongation of mouse survival in both local and disseminated xenograft mouse models. The antitumor activity of the anti-TfR1 antibodies was greater in an early stage of disease in the local model using 2F7-BR44 cells, as expected since tumor burden is lower at the time of treatment (starting on Day 2 after tumor challenge). In the bioluminescent imaging study of the local (s.c.) xenograft model using 2F7-BR44 cells, we could not detect metastases suggesting that these tumors may not be spontaneously metastatic when inoculated s.c. In this model, one treatment of hu128.1 starting when the tumor was palpable (~3–5 mm in diameter) showed similar antitumor activity compared to multiple treatments. This may be due to the high tumor burden at the initiation of treatment and/or a suboptimal dose or treatment schedules for the study. In the disseminated xenograft model using 2F7-BR44 cells, multiple treatments of hu128.1 did not increase the antitumor effect compared to one treatment. This may be explained by the brain metastatic capacity of these cells and the fact that our anti-TfR1 antibodies do not cross-react with mouse TfR1 [34] and thus do not cross the blood-brain barrier that blocks accessibility to brain tumors. However, in this disseminated model, imaging showed that hu128.1 can prevent metastases to the brain. Previous studies have shown that 2F7-BR44 cells grow within the brain, as evidenced by luminescence of isolated whole brains of mice systemically challenged with these cells, as well as by immunohistochemical studies of brain sections [29]. Importantly, the brain metastatic property of 2F7-BR44 cells is consistent with the reported high risk of primary and metastatic CNS involvement in HIV-infected individuals with NHL, which results in poorer survival [35,36,37]. The studies with the JB cell line show that this model can be inconsistent in that not all control mice develop tumors in every experiment. This is observed whether the cells are inoculated s.c. or i.v. Despite this fact, both ch128.1/IgG1 and hu128.1 prolonged survival of tumor-bearing mice in every experiment. 

The studies reported here were conducted in immunodeficient SCID-Beige mice. These mice have a limited immune cell repertoire in that they lack mature B and T cells due to the SCID mutation [38]. Thus, the adaptive immune response cannot contribute to the antitumor effects of ch128.1/IgG1 and hu128.1. In addition, these mice have an impairment in NK cell function due to the beige mutation [39,40]. Furthermore, these mice have impaired neutrophil activity [41], but maintain a functional macrophage population [39,40]. Our previous studies in xenograft models of MM, another B-cell malignancy, were also conducted in SCID-Beige mice and indicate that the mechanism of antitumor activity of our ch128.1 antibodies (both IgG1 and IgG3) is dependent on the Fc region of the antibodies. Mutant antibodies of ch128.1 that lack the ability to bind Fc gamma receptors (FcγRs) do not show antitumor activity [24,26]. Our previous studies also show that macrophages play an important role in these studies conducted in SCID-Beige mice [24]. Additionally, previous in vitro studies show that our ch128.1 antibodies can induce antibody-dependent cell-mediated cytotoxicity (ADCC) and antibody-dependent cell-mediated phagocytosis (ADCP) using either murine bone marrow-derived macrophages or human peripheral blood mononuclear cells (PBMC) in vitro [24,26,42]. Therefore, it is likely that the anti-NHL activity observed in these studies is also mediated through the induction of Fc-mediated effector functions induced by our anti-TfR1 antibodies. However, we cannot rule out other mechanisms of action, including iron deprivation caused by the sequestration of the TfR1 on the surface of NHL cells due to the simultaneous binding to FcγR on immune cells or the inhibition of iron uptake through other mechanisms. Further studies are needed to evaluate the mechanism of antitumor activity of ch128.1/IgG1 or hu128.1 in these xenograft mouse models of AIDS-NHL. It is also important to point out that in humans, the presence of a full repertoire of functional immune cells may further contribute to the antitumor effects of the anti-TfR1 antibodies, a possibility that needs to be evaluated in clinical trials.

TfR1 is expressed on many normal cells at low levels and also at higher levels on cells that are rapidly dividing and/or require large amounts of iron [16]. With the use of anti-TfR1 antibodies, toxicity to these normal cells expressing high levels of TfR1 is a concern. Previous studies show that antibodies containing the 128.1 variable regions bind the apical domain of TfR1 and do not inhibit the binding of transferrin to the receptor or block TfR1 internalization [21,22,34,43]. However, ch128.1/IgG1 partially blocks the binding of the heavy chain of ferritin (H-Ft) [43], known to bind the apical domain of TfR1 and play a role in the uptake of iron in erythroid progenitor cells [44,45,46]. It is important to note that H-Ft only binds TfR1 when expression levels are very high [45], which is not the case for most normal cells. Furthermore, since transferrin is the main iron importer in the majority of normal cells and ch128.1/IgG1 does not inhibit its binding or internalization, we expect the impact of ch128.1/IgG1 on iron uptake to be minimal in most normal cells. 

The most vulnerable cell population is hematopoietic erythroid progenitor cells that express high levels of TfR1 due to the large requirement of iron for hemoglobin synthesis [16,44,45]. As with many antibody-based therapeutics, our SCID-Beige mouse model is not meaningful to study toxicity to normal cells since the ch128.1 and hu128.1 antibodies do not cross-react with mouse TfR1 [34]. However, we have previously evaluated the potential toxicity of ch128.1/IgG1 on committed hematopoietic progenitor cells in an in vitro assay [43]. We have shown that only erythroid progenitor cells were affected by ch128.1/IgG1 treatment at high concentrations, as evidenced by a significant decrease in BFU-e (blast-forming units-erythroid) colonies [43]. It is important to note that treatment with ch128.1/IgG1 did not completely block BFU-e colony formation in this assay, even at concentrations up to 500 nM (75 μg/mL). For this assay, cells are incubated for 14 days in the presence of the antibody, conditions that may not adequately reflect those in a human or an animal model. Thus, these results may overestimate the toxicity of ch128.1/IgG1 on erythroid progenitors. CFU-GM (colony forming units-granulocyte macrophage) colony numbers were not affected by ch128.1/IgG1 treatment [43]. Toxicity to erythroid progenitors at high antibody concentrations is not surprising since these cells express the highest level of TfR1 in the body [44]. Our previous studies are consistent with a report from another group using the fully human anti-TfR1 IgG1 antibody JST-TfR09, also known as PPMX-T003, being evaluated in humans affected with the hematologic malignancy polycythemia vera (NCT05074550) [47]. This antibody blocks the binding of transferrin to TfR1 and has shown in vitro toxicity to human erythroblasts differentiated from CD34 hematopoietic stem cells at concentrations above 156 ng/mL [48]. Additionally, JST-TfR09 administered to cynomolgus monkeys (*Macaca fascicularis*) and to healthy human volunteers in a Phase I clinical trial induced moderate anemia [48,49,50]. Taken together, erythroid progenitor cells may be a vulnerable population for antibodies targeting TfR1 depending on the epitope targeted and whether they inhibit the binding of transferrin or H-Ft to the receptor. It is also important to mention that adverse effects (such as anemia) of antibodies, including those targeting TfR1, may be triggered by the effector functions of the Fc region such as ADCC and/or complement-mediated cytotoxicity (CDC) [16,51]. However, non-committed (pluripotent) hematopoietic stem cells are known to lack TfR1 expression [52,53,54]. In fact, previous studies using an immunotoxin consisting of an avidin fusion protein of ch128.1/IgG3 conjugated to a biotinylated form of the ribosomal inhibitory plant toxin saporin showed no effects on this cell population [55]. Thus, the toxic effects on erythroid progenitor cells, if present, is expected to be transient. Further studies using other animal models or even clinical trials are needed to more adequately address the potential toxicity of ch128.1/IgG1 or hu128.1.

## 5. Conclusions

In summary, our studies utilized xenograft mouse models using two different AIDS-BL cell lines, EBV-positive and EBV-negative. The use of the JB cell line in an in vivo model is in itself novel, and to our knowledge, this is the first report on the use of this cell line grown in mice to study the efficacy of anti-TfR1 antibodies. The use of the 2F7-BR44 variant cell line is also of importance since this is a very aggressive variant of this cell line that metastasizes to the brain. Importantly, ch128.1/IgG1 and hu128.1 are effective in these pre-clinical studies against very relevant forms of AIDS-NHL in both local and disseminated xenograft models. The results in this study suggest that the present anti-TfR1 antibodies, in particular hu128.1, should be further explored as a potential therapeutic for AIDS-NHL.

## 6. Patents

The following patent has been granted: No. PCT/US2011/031934; USSN: 13/639,172, U.S. Patent No. 8,734,799 (M.L.P. and T.R.D.-W.). Issued on 27 May 2014. Additionally, the following patent applications have been filed: No. PCT/US2020/059532 (M.L.P., P.V.C., J.C.A., and T.R.D.-W.), No. PCT/US 2021/023735 (M.L.P., O.M.-M., and T.R.D.-W.), and No. PCT/US2021/048714 (M.L.P., P.V.C., and T.R.D.-W.).

## Figures and Tables

**Table 2:** Median survival and statistical analysis for the data in the disseminated model of AIDS-NHL using 2F7-BR44 cells presented in Figure 3B.

Treatment	Number of Animals	Median Survival (Days)	*p*-Value Compared to IgG1	*p*-Value Compared to Buffer	*p*-Value Compared to hu128.1 (Day 2)
Buffer (Day 2)	9	23			
100 μg IgG1 (Day 2)	10	24		0.9024	
100 μg hu128.1 (Day 2)	10	42	<0.0001	<0.0001	
100 μg IgG1 (Days 2, 5, 8, 11)	10	25		0.5451	
100 μg hu128.1 (Days 2, 5, 8, 11)	10	39	<0.0001	<0.0001	0.1528

## Data Availability

The datasets generated during and/or analyzed during the current study are available from the corresponding author on reasonable request.

## Supplementary Materials

The following supporting information can be downloaded at: https://www.mdpi.com/article/10.3390/cancers15061816/s1, Figure S1: Individual tumor growth curves for the data shown in Figure 1A; Figure S2: Individual tumor growth curves for the data shown in Figure 1B; Figure S3: Individual tumor growth curves for the data shown in Figure 1C; Figure S4: Individual tumor growth curves for the data shown in Figure 2 for the bioluminescence imaging study; Figure S5: Individual tumor growth curves for the data shown in Figure 5A; Figure S6: Individual tumor growth curves for the data shown in Figure 5B; Figure S7: Comparison of buffer inoculation versus isotype negative control antibody treatment in the local model of AIDS-NHL using JB cells; Figure S8: Efficacy of ch128.1/IgG1 in the disseminated model of AIDS-NHL using JB cells.
