# Supplementary material for: Efficacy of Antibodies Targeting TfR1 in Xenograft Mouse Models of AIDS-Related Non-Hodgkin Lymphoma
Source: Cancers (Basel). 2023 Mar 17;15(6):1816. doi: 10.3390/cancers15061816 (PMC10046321; doi:10.3390/cancers15061816)
Supplement: Supplementary file 1 [file cancers-15-01816-s001.zip › cancers-2272594-supplementary.pdf]

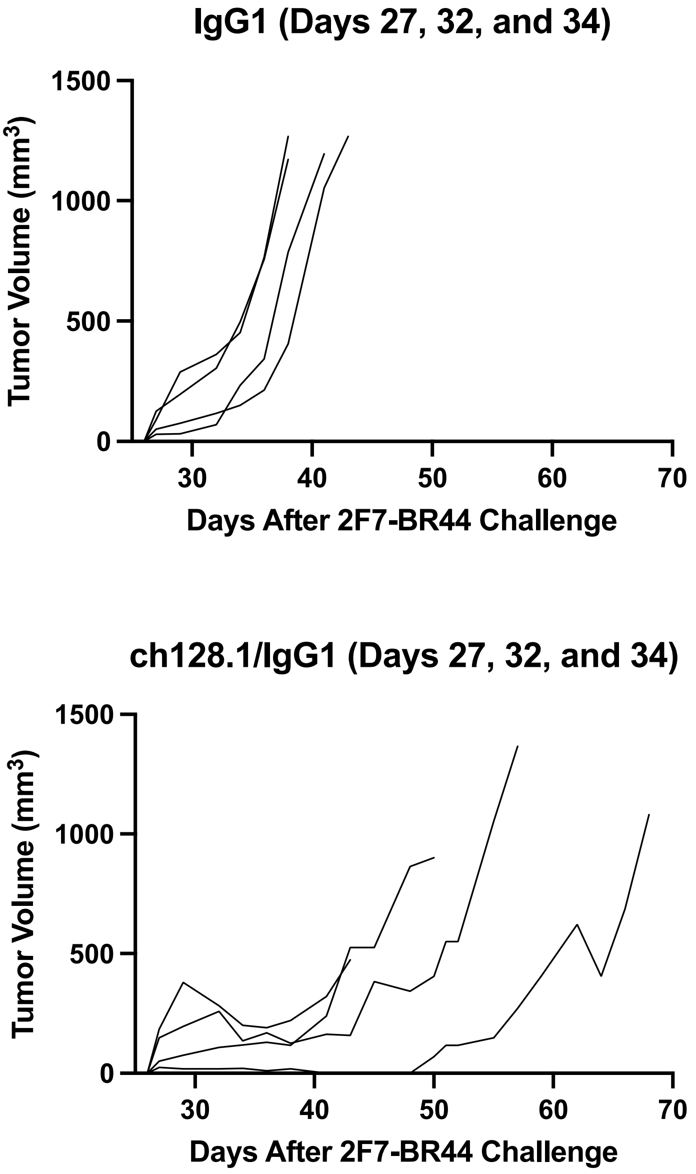

Figure S1. Individual tumor growth curves for the data shown in Figure 1A. SCID-Beige mice (8-12 weeks old) were challenged subcutaneously (s.c.) with 2 x 10^6 2F7-BR44 cells in the right flank. Once all mice developed palpable tumors (Day 27), mice were distributed by tumor size into two groups (*n* = 4 per group). Mice were treated intravenously (i.v.) with either 400 µg isotype negative control antibody (IgG1) or ch128.1/IgG1 as indicated. Individual tumor growth curves shown here correspond to the average tumor growth data shown in Figure 1A.

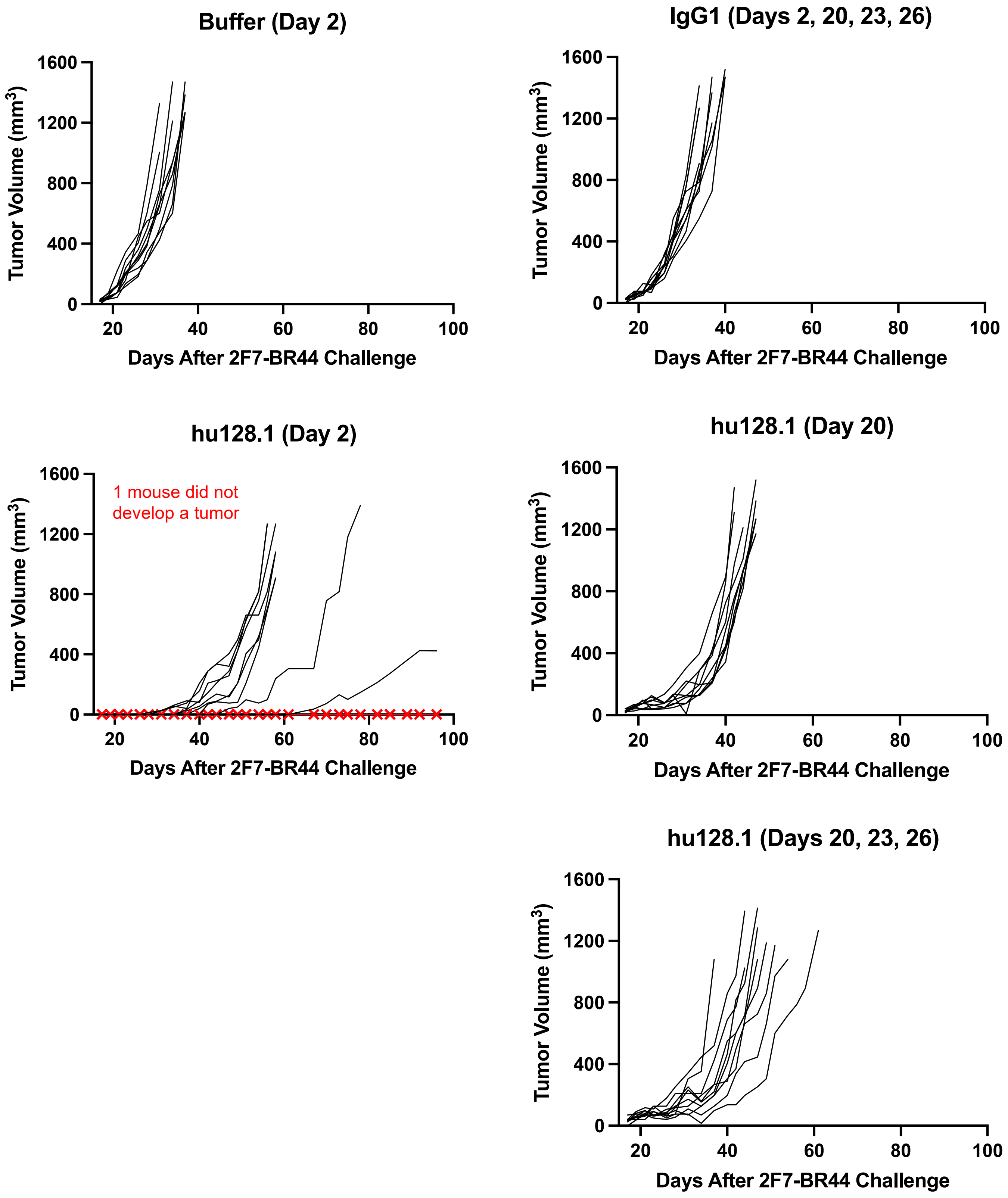

Figure S2. Individual tumor growth curves for the data shown in Figure 1B. SCID-Beige mice (8-12 weeks old) were challenged s.c. with 2 x 10^6 2F7-BR44 cells in the right flank. Mice were treated i.v. with 400 µg isotype negative control antibody (IgG1) or hu128.1 as indicated (*n* = 9 or 10 per group). Individual tumor growth curves shown here correspond to the average tumor growth data shown in Figure 1B.

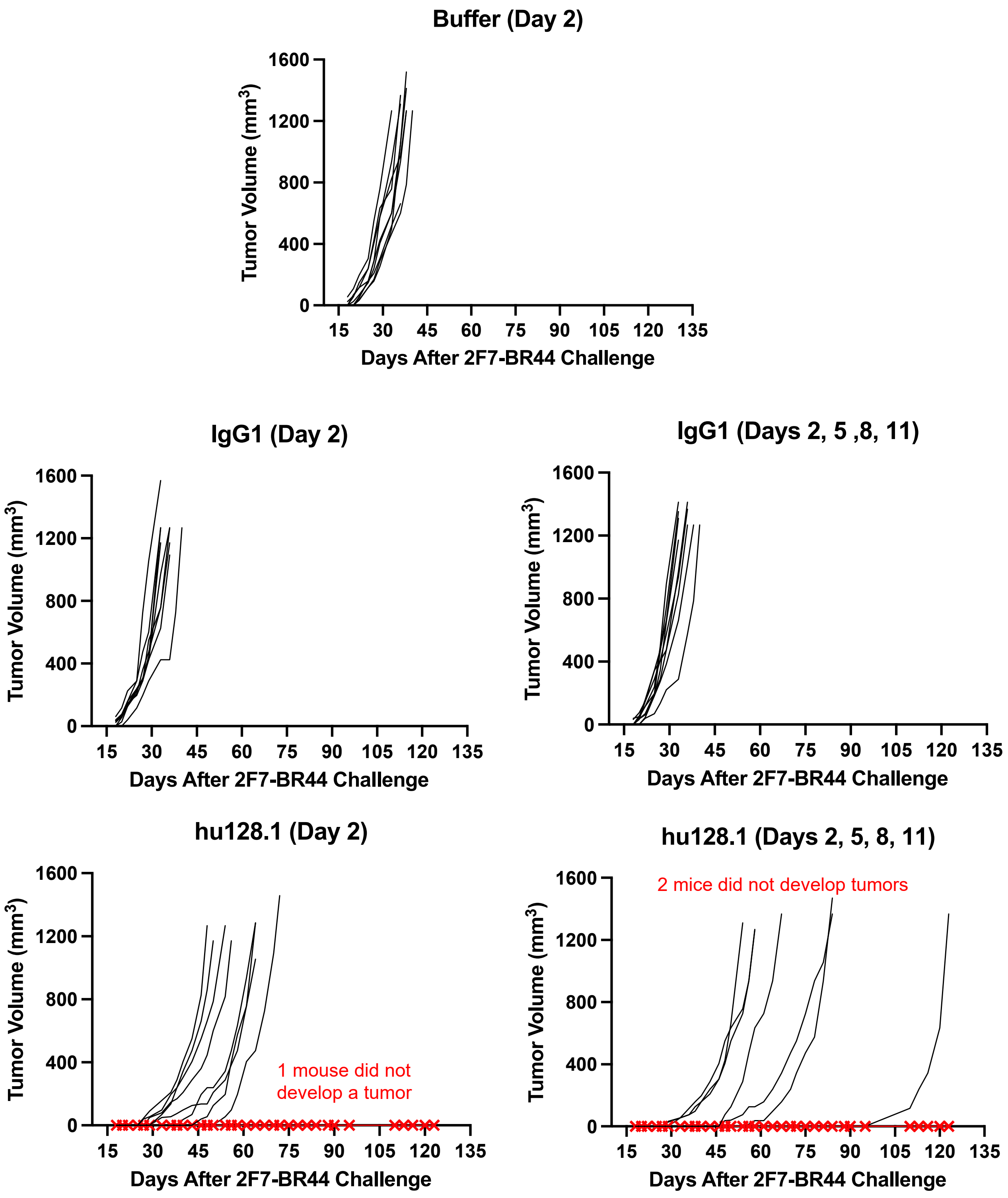

Figure S3. Individual tumor growth curves for data shown in Figure 1C. SCID-Beige mice (8-12 weeks old) were challenged s.c. with 2 x 10^6 2F7-BR44 cells in the right flank. Mice were treated i.v. with 400 µg isotype negative control antibody (IgG1) or hu128.1 as indicated (*n* = 9 per group). Individual tumor growth curves shown here correspond to the average tumor growth data shown in Figure 1C.

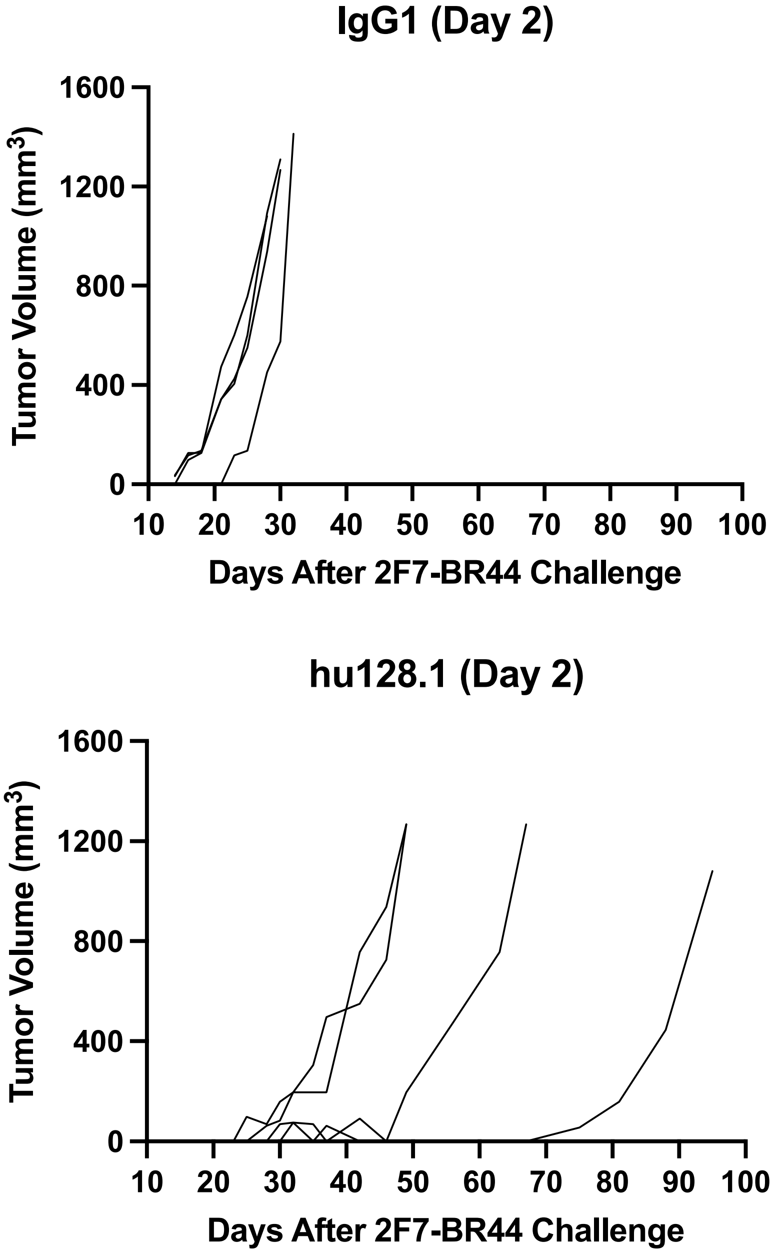

Figure S4. Individual tumor growth curves for the data shown in Figure 2 for the bioluminescence imaging study. SCID-Beige mice (8-12 weeks old) were challenged s.c. with 2 x 10^6 2F7-BR44 cells in the right flank. Mice were treated i.v. on Day 2 with 400 µg isotype negative control antibody (IgG1) or hu128.1 (*n* = 4 per group). Individual tumor growth curves shown here correspond to the average tumor growth data shown in Figure 2.

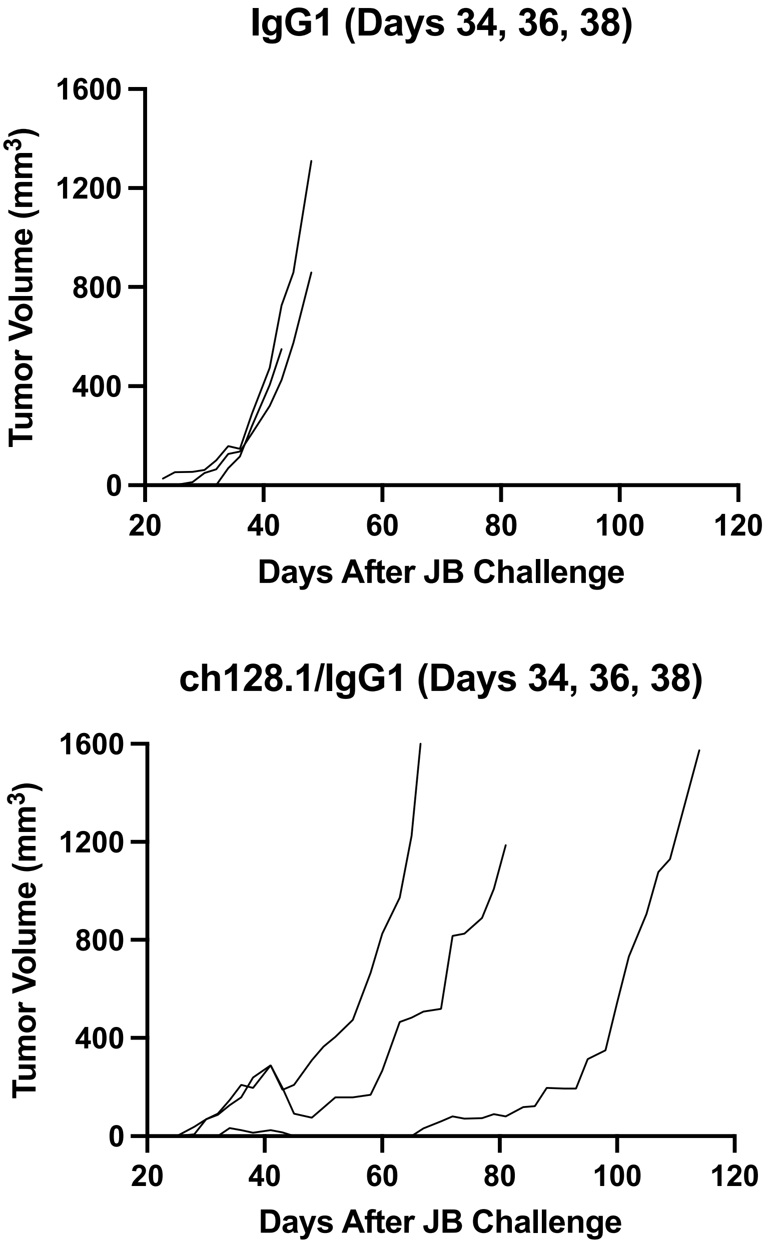

Figure S5. Individual tumor growth curves for the data shown in Figure 5A. SCID-Beige mice (8-12 weeks old) were challenged s.c. with 5 x 10^6 cells in the right flank. Once all mice developed palpable tumors (Day 34), mice were distributed by tumor size into two groups (n = 3 per group). Mice were treated i.v. with either 400 µg isotype negative control antibody (IgG1) or ch128.1/IgG1 as indicated. Individual tumor growth curves shown here correspond to the average tumor growth data shown in Figure 5A.

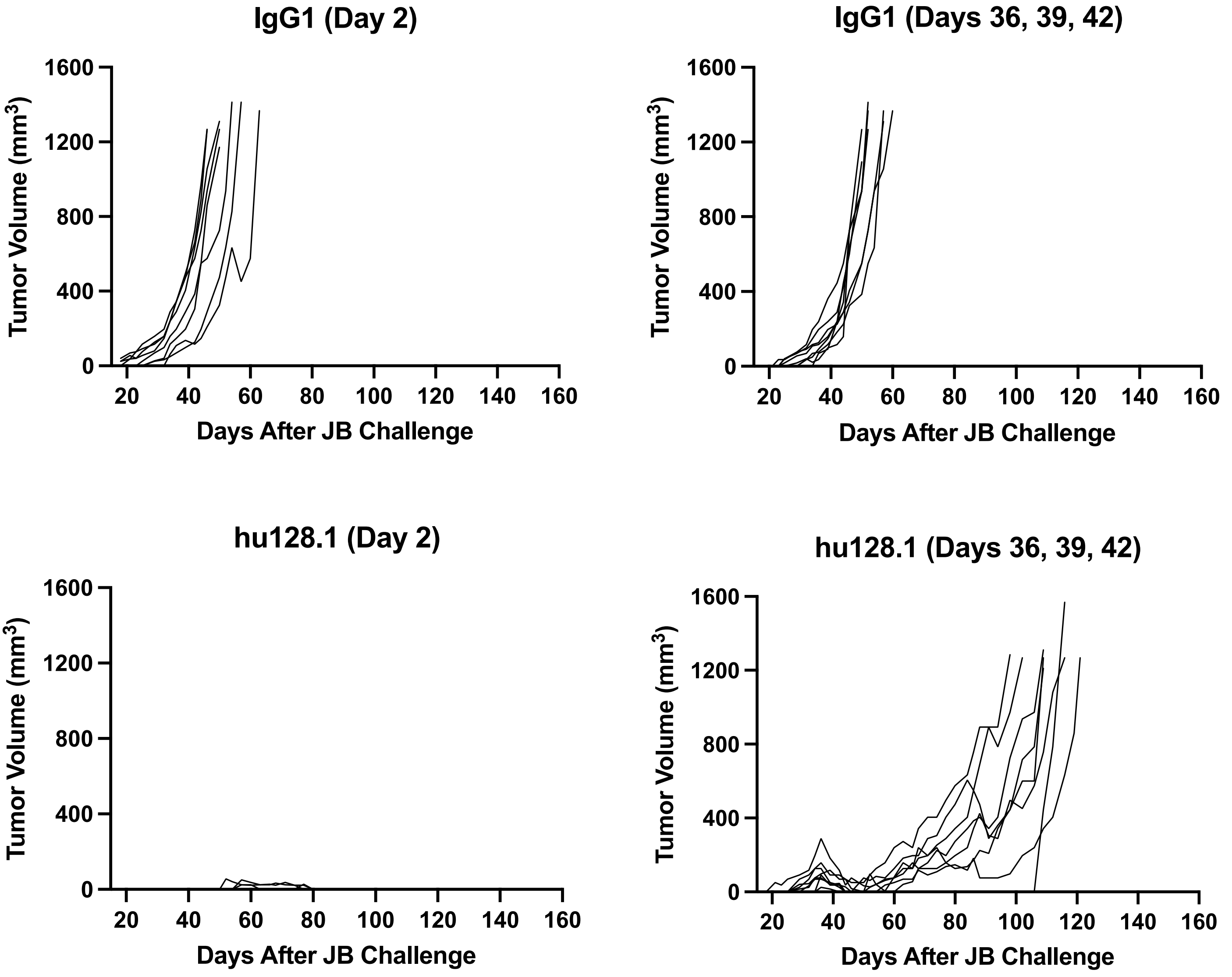

Figure S6. Individual tumor curves for the data shown in Figure 5B. SCID-Beige mice (8-12 weeks old) were challenged s.c. with 5 x 10^6 cells in the right flank. Mice were treated i.v. with 400 µg isotype negative control antibody (IgG1) or ch128.1/IgG1 as indicated. Individual tumor growth curves shown here correspond to the average tumor growth data shown in Figure 5B.

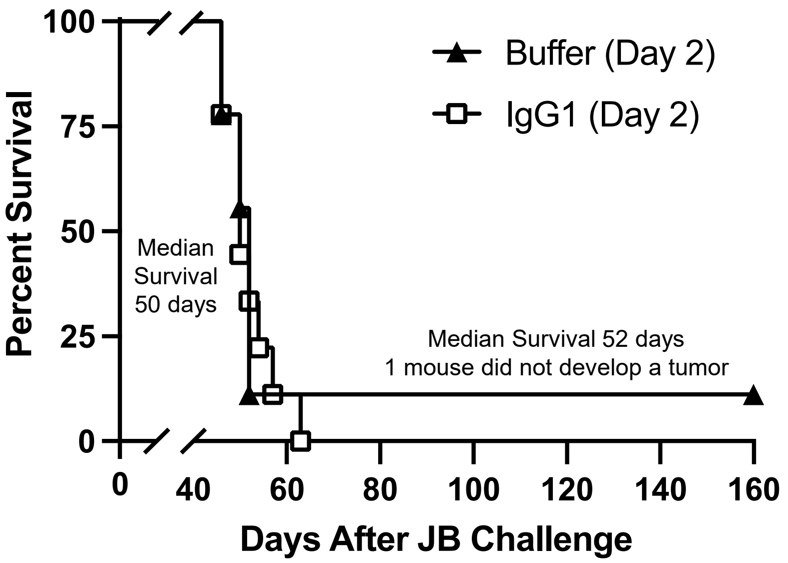

Figure S7. Comparison of buffer inoculation versus isotype negative control antibody treatment in the local model of AIDS-NHL using JB cells. SCID-Beige mice (8-12 weeks old) were challenged s.c. with 5 x 10^6 cells in the right flank. Mice were treated i.v. with 400 µg isotype negative control antibody (IgG1) or inoculated with buffer alone on Day 2 after tumor implantation (n = 9). The Kaplan-Meier survival plot is shown with the median survival for each treatment group. No significant survival difference was observed between these two negative control groups. The data shown for the isotype negative control antibody (IgG1) are the same data presented in Figure 5B. The treatments presented in Figure 5B along with the group inoculated with buffer alone were conducted in the same study.

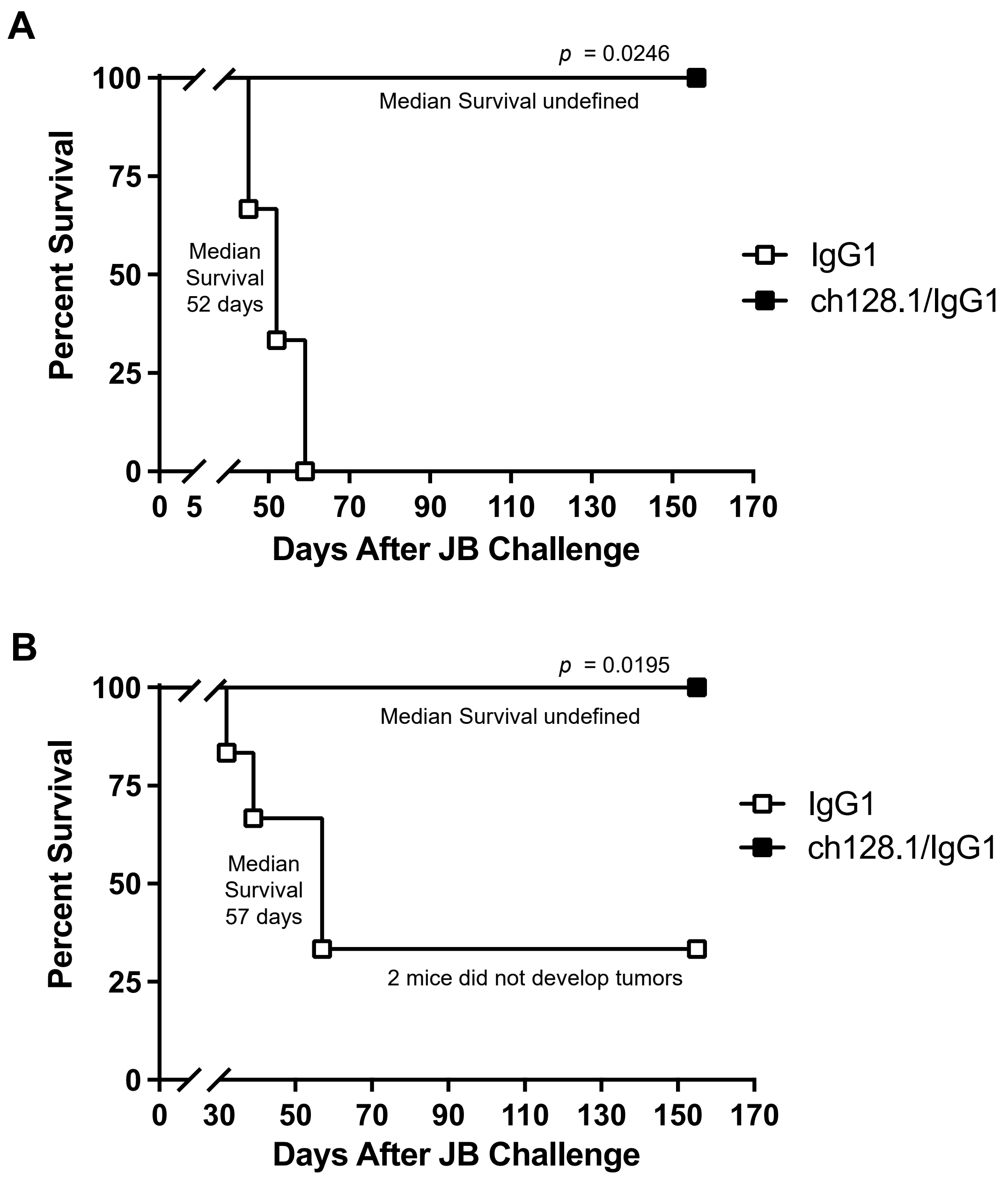

Figure S8. Efficacy of ch128.1/IgG1 in the disseminated model of AIDS-NHL using JB cells. SCID-Beige mice (8-12 weeks old) were challenged i.v. with 5 x 10^6 cells via the tail vein. Mice were treated i.v. on Day 2 with 100 µg isotype negative control antibody (IgG1) or ch128.1/IgG1 (A) $n = 3$ or (B) $n = 6$ in two independent experiments. The Kaplan-Meier survival plots are shown with the median survival for each treatment group.
